# Urethral injury during vaginal delivery: Incidence, management and long‐term outcomes

**DOI:** 10.1002/ijgo.70941

**Published:** 2026-03-27

**Authors:** Yoav Baruch, Ronen Gold, Yariv Stabholz, Michael Lavie, Yariv Yogev, Asnat Groutz

**Affiliations:** ^1^ Lis Maternity and Women's Hospital, Tel Aviv Sourasky Medical Center, Gray Faculty of Medical and Health Sciences Tel Aviv University Tel Aviv Israel

**Keywords:** BFLUTS, MESA, Obstetric‐associated urethral injury, USIQ, vacuum‐assisted delivey, vaginal delivery

## Abstract

**Objective:**

This study examines the incidence, obstetric risk factors, surgical management, and long‐term outcomes of obstetric‐associated urethral injuries during vaginal deliveries.

**Methods:**

A retrospective case series was conducted at a single, tertiary university‐affiliated medical center between January 2018 and December 2023. Demographic, obstetric, and surgical data were retrieved from the hospital's electronic records. Validated symptom questionnaires (BFLUTS, USIQ, and MESA) were administered prospectively at ≥18 months postpartum.

**Results:**

Of 59 553 vaginal deliveries, five urethral injuries were identified, equivalent to 0.83 per 10 000 vaginal deliveries. Injuries comprised urethral tear or complete detachment of the urethra. One injury followed vacuum‐assisted delivey, one occurred during precipitated labor, and three during uncomplicated spontaneous vaginal deliveries. Two patients also exhibited significant symphyseal diastasis. Four cases were diagnosed immediately after delivery, and one other case was diagnosed several hours after delivery due to anuria. All cases were managed in the operating room by a senior urologist. At a mean follow‐up of 42 months (range 18–83), questionnaire scores were low (BFLUTS 2.0 [1.0–16.5], USIQ 0 [0–37.5], MESA stress ratio 14.1 [2.35–94.1]). No patients reported other long–term sequelae associated with urethral trauma, such as urethral stricture or urethrovaginal fistula. At follow‐up, two patients had given birth again; both underwent uncomplicated vaginal deliveries.

**Conclusion:**

Urethral injury during vaginal delivery is rare. Early diagnosis and immediate surgical repair were associated with favorable long‐term lower urinary tract symptoms. Concomitant symphyseal diastasis in two patients may suggest clinical association. Further studies are needed to establish management guidelines.

## 1 INTRODUCTION

Obstetric‐associated lower urinary tract injuries might occur during vaginal deliveries but are far more common during cesarean delivery, with reported incidence of 0.02%–0.94%.^1^, ^2^, ^3^, ^4^ Urethral trauma during vaginal delivery is an uncommon yet potentially debilitating obstetric complication. Although peri‐urethral lacerations are frequently encountered after delivery, full thickness urethral tears or urethral detachment requiring repair are extremely rare, with reported incidence of 0.002%.^1^

Several risk factors for lower urinary tract injury during vaginal delivery have been reported. Instrumental deliveries subject the perineal body and urethra to tangential shear forces.^5^ A previous cesarean section is another risk factor as adhesions of the bladder wall might render it more prone to injury when exposed to the shearing force during vaginal delivery.^4^ Prolonged second stage and obstructed labor might result in sustained compression of the fetal head against the maternal pubic symphysis, potentially leading to ischemic injury of the urethral and bladder‐neck vascular beds. Conversely, precipitate labor affords insufficient time for gradual tissue distension, predisposing to potential urethral damage.^5^ Delivery with a Foley catheter in situ during the second stage of labor was reported as an additional risk factor for lower urinary tract injury.^6^

Animal models elucidate the possible underlying pathophysiology. Damaser et al. demonstrated 50% reductions in peri‐urethral blood perfusion under controlled vaginal distension, producing local hypoxia and structural disruption.^7^ Song et al. found persistent denervation of the external urethral sphincter 9 weeks after simulated childbirth injury, despite normalization of leak‐point pressure.^8^ These animal studies point to two plausible mechanisms for obstetric‐related urethral injury: impaired neural integrity and diminished blood reperfusion.

Possible complications of urethral trauma include urethral stricture, urethrovaginal fistula, chronic pelvic pain, and stress or urgency urinary incontinence.^9^, ^10^ Guidance on management of obstetric‐associated urethral trauma is drawn largely from literature regarding synthetic mid‐urethral slings and pelvic fractures.^11^

The 2023 European Association of Urology trauma guidelines recommend definitive urethral repair within 7 days to minimize complications; while early repair is recommended when feasible.^12^ To date, no evidence‐based guidelines exist for the optimal management of urethral injury during vaginal delivery. Published literature is largely limited to isolated case reports, or small institutional series.^13^, ^14^, ^15^, ^16^

We therefore aimed to (i) establish the incidence of obstetric‐associated urethral injury, (ii) describe obstetric circumstances and surgical approaches, and (iii) evaluate long‐term symptoms using validated questionnaires.

## 2 METHODS

A retrospective case series of obstetric‐associated urethral injuries occurring between 2018 and 2023 was conducted at a single tertiary, university‐affiliated medical center with over 13 000 deliveries annually. Prospective clinical outcome assessment was performed in January 2025, ensuring a minimum follow‐up duration of 18 months for all included cases. Urethral injuries were defined as urethral tears involving the urethral wall, as well as urethral detachments. Superficial peri‐urethral lacerations without urethral wall involvement were excluded. Demographic, obstetric, and surgical data were retrieved from the hospital's electronic records. Validated symptom questionnaires were administered prospectively at ≥18 months postpartum. The medical center serves a predominantly local white population. Primiparous parturients constitute approximately 50% of all deliveries. The cesarean section rate is approximately 22–23% and vacuum‐assisted deliveries account for 11% of all vaginal births. Uncomplicated deliveries are managed by midwives, whereas all instrumental deliveries (performed exclusively with vacuum) are carried out by obstetricians. Routine perineal protection is applied during delivery, and medio‐lateral episiotomy is performed selectively. The overall incidence of episiotomies during the study period was 19.1%. Urethral injuries were surgically repaired by senior urologists. All women were initially scheduled for follow‐up in the urology clinic, as all repairs were performed by the urology team. Subsequently, after completion of the puerperium, patients were referred for continued follow‐up in the hospital's urogynecology clinic. Additionally, all women were advised to begin pelvic floor muscle training within the first month after delivery.

Data of all vaginal deliveries between January 2018 and December 2023 were retrieved from the hospital's computerized database. Cases were identified based on the ICD‐9 diagnosis of “Other injury to pelvic organs, with delivery” (code 665.5). In addition, we reviewed our ward's mandatory incident‐reporting system, which captures all unusual or complex genital tract injuries. These events are systematically reported, documented, and reviewed in accordance with institutional policy to ensure quality of care. Extracted data included demographic variables, personal medical history, obstetric variables, urethral injury details, and intraoperative management. Prolonged second stage was defined as 2 h or more for nulliparous and 1 h or more for parous women, with an additional 1 h for women under epidural anesthesia.

In January 2025, all patients who sustained obstetric‐associated urethral injury completed three validated questionnaires through a prospective telephone interview: (1) BFLUTS (Bristol Female Lower Urinary Tract Symptoms Questionnaire), with 19 items covering filling, voiding, incontinence, sexual function, and quality‐of‐life domains, each scored 0–4, and higher scores indicating worse symptoms^17^; (2) USIQ (Urgency Severity and Impact Questionnaire), with seven items measuring urinary urgency severity, urinary frequency, and impact on daily activities, total score 0–100, and higher scores reflecting greater bother^18^; and (3) MESA (Medical, Epidemiologic, and Social Aspects of Aging Stress/Urge Incontinence Questionnaire) composed of 15 dichotomous items, including six questions for urgency urinary incontinence (UUI) and 9 for stress urinary incontinence (SUI) that allows the generation of separate subscores for stress and urge symptoms. A specific stress‐score ratio was then used to differentiate between stress‐predominant and urgency‐predominant urinary leakage.^19^

Given that only five cases were identified, statistical evaluation was confined to descriptive analyses. The study was approved by the institutional review board. Verbal informed consent was obtained at the time of telephone contact prior to administering the questionnaires. Participation was entirely voluntary, and patients could decline or opt out at any stage.

## 3 RESULTS

A total of 59 553 vaginal deliveries were recorded over the 6‐year study period, of which 6905 (11.6%) were vacuum‐assisted. Five cases of obstetric‐associated urethral injuries were identified, reflecting an overall incidence of 0.83 per 10 000 vaginal deliveries. Except for one case, all cases were identified immediately after delivery.

Demographic data are presented in Table 1. Mean maternal age was 31.6 years (28–38 years). Body mass index values ranged from 18.8 to 30.4 kg/m^2^. Three of the five patients were nulliparous. One participant had a history of ulcerative colitis and was treated with mesalazine. None of the patients were smokers.

**TABLE 1:** Demographic and clinical characteristics.

Case	Age (y)	Ethnicity	BMI (kg/m^2^)	Comorbidity	Previous surgery	Medications	Previous deliveries
1	38	White	n/a	None	1. Appendectomy 2. Laparotomy due to bowel obstruction	None	0
2	28	White	19.4	None	1. Vesicoureteral reflux repair	None	One uncomplicated vaginal delivery (3004 g)
3	32	White	30.4	None	None	None	One vacuum‐assisted delivery (2970 g)
4	30	White	18.8	Ulcerative colitis	None	Mesalazine	0
5	30	White	26.9	None	None	None	0

Abbreviation: BMI, body mass index.

Obstetrical and intrapartum data are summarized in Table 2. One urethral injury followed vacuum‐assisted delivery for prolonged second stage, one occurred during precipitated labor, and three others during normal vaginal deliveries. Birthweights ranged between 2560 and 3470 g.

**TABLE 2:** Intrapartum characteristics (*n* = 5).

Case	P	GA	Labor onset	Epidural	PSS (second stage duration)	Mode of delivery	Episiotomy	Perineal tears	Birth weight (g)
1	0	39 + 6	Spontaneous	Yes	No (Precipitated)	Vaginal delivery	No	No	2970
2	1	38 + 0	Augmentation with oxytocin due to PROM	Yes	No (49 min)	Vaginal delivery	No	First degree	3470
3	1	40 + 1	Spontaneous	No	No (64 min)	Vaginal delivery	No	First degree	3466
4	0	38 + 1	Spontaneous	No	Yes (232 min)	Vacuum‐assisted delivery	Yes	No	2560
5	0	40 + 2	Spontaneous	No	Yes (195 min)	Vaginal delivery	No	Second degree	2925

Abbreviations: GA, gestational age at delivery in (weeks); P, parity; PROM, premature rupture of membranes; PSS (min), prolonged second stage.

Urethral injury characteristics and surgical management are presented in Table 3. All tears involved the distal urethra (mean length 2.4 cm). The first case was previously reported in detail^16^: a 38‐year‐old primigravida at term underwent a precipitated labor without prior bladder catheterization and sustained an 8‐cm longitudinal tear that extended from the distal urethra into 4 cm of the anterior bladder mucosa, leading to detachment of the bladder‐neck from its peri‐urethral attachments. The tear was surgically repaired in two layers with interrupted 3‐polyglactin sutures. An intraoperative methylene‐blue leak test was negative, and cystoscopy confirmed intact repair. Concomitant symphyseal diastasis (1.8 cm) was also noted, and the patient required assisted ambulation for the first 2 weeks postpartum. Analgesia and physiotherapy were initiated, with gradual mobility improvement.

**TABLE 3:** Urethral injury: Characteristics and management.

Case	Urethral tear length (cm)	Bladder involvement	Timing of repair	Repair	Sutures	Antibiotics	Catheter (days)
1	4	Yes	Immediate	Layered	3–0 Polyglactin	Amoxicillin–clavulanate 875 mg PO BID × 7 days	21
2	4	No	Immediate	Layered	3–0 Polyglactin	Amoxicillin–clavulanate 875 mg PO BID × 7 days	14
3	1	No	Immediate	Layered	4–0 Polyglactin	Amoxicillin–clavulanate 875 mg PO BID × 7 days	7
4	0.5	No	12 h postpartum	None. Catheterization only	None	Ampicillin and gentamicin IV × 48 h	10
5	Detachment	No	Immediate	Layered	2–0 Polyglactin	Ceftriaxone and metronidazole IV × 7 days, amoxicillin–clavulanate 875 mg PO BID × 5 days	5

The second case of urethral injury occurred during a vacuum‐assisted deliveriy for prolonged second stage. A 4‐cm urethral tear was repaired trans‐vaginally with polyglactin 3–0, and a 14F Foley was left for 14 days. A 7‐day course of prophylactic oral amoxicillin–clavulanate was administered.

The third case was diagnosed immediately after spontaneous delivery. Inspection revealed a 1‐cm urethral tear, and management consisted of layered clusore using 4–0 polyglactin sutures, followed by 7 days of continuous bladder drainage with a 16F Foley catheter, as well as oral amoxicillin–clavulanate therapy.

The fourth case was diagnosed 12 h postpartum due to anuria and elevated serum creatinine (1.4 mg/dL). The patient promptly transferred for computed tomography (CT) urography, which confirmed a urinoma. Cystoscopy revealed a small full‐thickness urethral perforation leading to the peritoneal cavity. The bladder was essentially intact except for superficial bladder‐neck lacerations. No suturing was performed, and treatment included continuous bladder drainage with an 18F council‐tip catheter for 10 days and prophylactic intravenous ampicillin–gentamicin regimen for 48 h.

The fifth case was of a proximal urethral detachment without a discrete urethral tear. Examination under anesthesia revealed complete mobility of the urethra, which was detached from the anterior vaginal wall, thereby rendering the periosteal layer of the symphysis pubis visible. Management consisted of a two‑layer repair using 2‑0 polyglactin sutures and five days of bladder drainage with an 18F Foley catheter. Prophylactic antibiotic therapy included seven days of intravenous ceftriaxone and metronidazole, followed by a five‑day course of oral amoxicillin–clavulanate. To exclude a pubic bone fracture, a pelvic CT scan was performed, which demonstrated a 3.4‐cm symphyseal diastasis without evidence of fracture.

Results of long‐term functional outcomes of the five patients are presented in Table 4. Mean follow‐up was 42 ± 22 months (range 18–83). Overall, questionnaire scores were low (BFLUTS 2.0 [1.0‐16.5], USIQ 0 [0‐37.5], MESA stress ratio 14.1 [2.35‐94.1]). The validated questionnaire scores showed minimal residual morbidity in four of the five patients. Quality‐of‐life domains were uniformly zero or near‐zero in three of these patients. The first patient, who sustained the combined 8 cm urethro‐vesical tear, accounted for most of the symptom burden recorded in Table 4. Her BFLUTS incontinence subscore was 10, she reported six SUI and 12 UUI episodes per week, and her MESA stress‐score ratio reached 66.7%. Her USIQ impact was moderate, indicating that the symptoms had an important effect on daily activities. No patient reported symptoms that might raise the suspicion of fistula, or urethral stricture. Two patients (Case 4 and 5) subsequently underwent uncomplicated spontaneous vaginal deliveries at other institutions. No specific recommendations regarding mode of delivery were given and the delivery proceeded without maternal or neonatal complications. The remaining three patients have not conceived since their index births.

**TABLE 4:** Long‐term patient‐reported outcomes.

	Case 1	Case 2	Case 3	Case 4	Case 5
Follow‐up (months)	83	46	33	18	34
Bristol Female Lower Urinary Tract Symptoms questionnaire (BFLUTS)					
Filling symptoms	6	1	1	1	0
Voiding symptoms	1	0	0	0	0
Urinary incontinence	10	0	0	2	1
Sexual function	6	0	0	0	0
Quality of life	6	0	1	0	0
Medical, Epidemiologic, and Social aspects of Aging (MESA) questionnaire					
Stress total score	18	0	3	1	1
Stress score ratio	66.7	0	11.1	3.7	3.7
Stress urinary incontinence (episodes per week)	6	0	0	0	0
Total urgency score	13	0	0	3	0
Urgency score ratio	48.1	0	0	16.7	0
Urgency urinary incontinence (episodes per week)	12	0	0	0	0
Urgency, Severity and Impact Questionnaire (USIQ)					
Symptom severity	37.5	0	0	0	0
Related quality of life	75	0	0	10	0

## 4 DISCUSSION

### 4.1 Main findings

Obstetric‐associated urethral injury is rare in modern obstetric practice. Results of the present study show that urethral injury occurred in fewer than one per 10 000 vaginal deliveries. At long‐term follow‐up, most patients demonstrated favorable urinary outcomes. We observed a possible clinical association with symphyseal diastasis, warranting further investigation.

### 4.2 Results in the context of previous studies

Our incidence aligns with prior small institutional reports and underscores the rarity of the complication when modern obstetric and perineal protective techniques are applied. Of the five cases presented in the current case series, one followed a vacuum‐assisted delivery. Given that vacuum‐assisted deliveries accounted for 11.5% of vaginal births in our cohort, this might support previous evidence that instrumental deliveries might increase the risk of lower urinary tract trauma. The solitary case of urethral injury following precipitate labor might support biomechanical theories that rapid fetal descent precludes gradual tissue stretching and the risk for severe perineal and pelvic damage.^5^ Interestingly, three of the five injuries occurred during otherwise uncomplicated spontaneous deliveries. One patient had a history of surgery for vesicoureteral reflux, and another had longstanding ulcerative colitis; both conditions are recognized risk factors for pelvic adhesions and might, in turn, increase susceptibility of the urethra to avulsive forces during childbirth.

One of the cases (case 4) was diagnosed following anuria and urinoma. The proximal urethra and bladder neck are positioned immediately posterior to the inferior margin of the symphysis pubis, and, theoretically, a full‐thickness tear at the 12‐o'clock position can extend cranially just below the bladder neck into the retropubic (Retzius) prevesical space. An alternative possible mechanism is that the laceration extended through the inferior urethral wall into the anterior cul‑de‑sac, thereby creating a direct communication with the intraperitoneal cavity.

In the present series, two of five patients (40%) with urethral trauma also demonstrated marked symphyseal diastasis. Although this coexistence is rarely described,^13^ its association with obstructed labor suggests a shared biomechanical mechanism. We propose a hypothetical pathway in which excessive intrapartum shear forces might both separate the symphysis and exert traction on peri‐urethral tissues, potentially explaining the concomitant proximal urethral detachment observed. Whether pre‐existing symphysis laxity predisposes to urethral injury, or whether the diastasis represents a secondary response to extreme intrapartum forces, remains uncertain.

Early orthopedic assessment, imaging to quantify diastasis, as well as early physiotherapy are therefore recommended in patients presenting obstetric‐associated urethral injury. Further multicenter data are needed to define the true incidence of combined injuries and to clarify whether symphyseal diastasis increases the risk of acute or long‐term urethral dysfunction.

In the present series, all urethral repairs were performed by board‐certified consultant urologists. There was a wide heterogeneity in both suture material and postoperative catheterization strategy, highlighting the lack of guidelines for urethral repair in the setting of obstetrical injury. Within this small series, surgeons selected 2–0, 3–0, or 4–0 polyglactin sutures for layered closure, whereas in one case no suturing was performed. There is no evidence‐based consensus regarding the optimal suture material. Similarly, continuous bladder catheterization ranged from 5 days (for the case with no true urethral tear but rather a displacement) to 21 days in the case with a urethral tear extending into the bladder wall. Between these extremes, dwell times of 7–14 days were chosen for intermediate‐sized, bladder‐sparing urethral tears. Such variability reflects the paucity of evidence‐based recommendations: while shortening the duration of catheterization might reduce patient discomfort and decrease catheter‐associated urinary‐tract infections, premature catheter removal can compromise urethral healing. None of the patients experienced catheter‐related infection or erosion. In addition, the patients received heterogeneous prophylactic antibiotic regimens, varying in both agents and duration, highlighting the lack of prophylactic guidelines. None of the patients required blood transfusion at any point during hospitalization or follow‐up. Validated symptom scores at a mean 42 month follow‐up confirmed minimal residual morbidity. These findings support early surgical intervention and short to intermediate catheterization as an effective strategy for isolated obstetric‐associated urethral injuries. The use of telephone‐administered questionnaires might introduce interviewer and social‐desirability biases; however, this approach also reduces the response and recall biases commonly associated with mailed surveys. Women with more severe symptoms might be more inclined to participate in follow‐up, and conducting interviews by telephone likely mitigated non‐response bias and minimized missing data. These advantages are particularly important in a small cohort, where maximizing follow‐up completeness is essential for the robustness of the findings.

### 4.3 Clinical implications

Prenatal counseling after prior obstetric‐associated urethral injury remains challenging as no guidelines currently address the safest mode of delivery for women who sustained urethral injury in a previous delivery. Whether a repeated vaginal delivery carries a meaningful risk of recurrent urethral or bladder trauma remains unknown. Elective cesarean section could theoretically reduce shear forces at the ureterovesical junction and thus prevent recurrence of the injury. However, it introduces surgical morbidity in current or future pregnancies.

An analogous scenario is the management of complex obstetric anal sphincter injury. Given shared mechanisms and risk factors, clinicians should weigh elective cesarean delivery to minimize long‐term risk, despite inherent uncertainty.

Until prospective data becomes available, decisions should be individualized. Clinical factors to consider include the length and complexity of the urethral tear, bladder involvement, residual urinary symptoms, and pelvic‐floor imaging if available. Shared decision‐making must acknowledge the paucity of evidence and balance the possible yet unquantified risk of recurrent urethral damage against the known risks of cesarean delivery.

### 4.4 Strengths and limitations

To the best of our knowledge, the present series is the biggest case series from a single center of obstetric‐associated urethral injury. A key strength of this study is the use of prospective validated questioners for long‐term outcomes, unlike previous reports. Study limitations include the small sample size and the fact that the reported prevalence is based solely on diagnosed cases. Consequently, the true incidence might be underestimated if additional urethral injuries remain undetected. Additionally, given the heterogeneity in surgical management, this small series does not allow any conclusions to be drawn regarding optimal treatment. An additional limitation is the absence of forceps‐assisted deliveries in our cohort, which restricts the generalizability of our findings to settings where forceps use is more common. A further limitation is the lack of post‐repair urodynamic testing or cystoscopy. Without these objective assessments, we cannot fully characterize urethral healing and lower urinary tract function.

By providing robust follow‐up data, we hope to provide information for future protocols and highlight areas requiring multicenter collaboration. Long‐term follow‐up data from other investigations are essential for developing evidence‐based guidelines and standardizing care and might improve outcomes in this rare but significant obstetric complication.

## 5 CONCLUSION

Urethral injury during vaginal delivery is rare but might be clinically substantial. Prompt recognition and early surgical repair, followed by short‐to‐intermediate continuous transurethral catheter drainage, were associated with favorable long‐term functional outcomes in cases of isolated urethral injury. Clinicians should maintain vigilance for postpartum urethral bleeding, anuria, or difficult catheterization, particularly after instrumental or precipitous vaginal deliveries. Cystoscopy can confirm tear extent, and early urologic involvement is critical. Concomitant symphyseal diastasis in two patients may suggest clinical association. Establishing multicenter registries will be pivotal for formulating definitive management guidelines.

## AUTHOR CONTRIBUTIONS

**Yoav Baruch:** Conceptualization, data curation, investigation, writing – original draft. **Ronen Gold:** Review and editing. **Yariv Stabholz:** Review and editing. **Michael Lavie:** Data curation, review and editing. **Yariv Yogev:** Writing – validation, review and editing. **Asnat Groutz:** Supevision, review and editing. All authors revised the article for important intellectual content and approved the final version submitted for publication.

## FUNDING INFORMATION

This research did not receive any specific grant from funding agencies in the public, commercial, or not‐for‐profit sectors.

## CONFLICT OF INTEREST STATEMENT

The authors declare no conflicts of interest.

## ETHICS STATEMENT

This retrospective analysis was approved by the Institutional Review Board (Approval No. TLV‐0052‐23) and conducted in accordance with the principles of the Declaration of Helsinki.

## Data Availability

The datasets generated and/or analyzed during the current study are available from the corresponding author on reasonable request.
